# Genome Assembly of Schlumbergera Virus X Infecting Prickly Pear (Opuntia cochenillifera) in Brazil

**DOI:** 10.1128/genomeA.00133-15

**Published:** 2015-03-19

**Authors:** Márcio Martinello Sanches, Natalia Silva Lamas, Marcelo Banho A. Reis, Juan Gabriel Arieta-Sosa, Eduardo Romano, Fernando Lucas Melo, Simone Graça Ribeiro

**Affiliations:** a Embrapa Recursos Genéticos e Biotecnologia, Brasilia, Brazil; b Universidade de Brasília, Brasília, Brazil

## Abstract

Potexviruses frequently infect plants from the family Cactaceae. We report the complete genome sequence of a new Schlumbergera virus X (SchVX) isolate. The genome has 6,615 nucleotides and shares 94% nucleotide identity with SchVX-K11 from Schlumbergera. This is the first sequence of an isolate of SchVX from the genus Opuntia*.*

## GENOME ANNOUNCEMENT

Prickly pear (Opuntia cochenillifera) (family Cactaceae) is a valuable forage for livestock feeding during the dry season in the semiarid northeast region of Brazil. It contains about 90% water and is a liquid supplement, especially for dairy cattle, goats, and sheep (1, 2). Because of their vegetative propagation, cactaceous plants are often infected by viruses, especially potexviruses.

The potexviruses (family *Alphaflexiviridae*, genus *Potexvirus*) are rod-shaped, nonenveloped, single-stranded, positive-sense RNA viruses (3). Four potexviruses infecting cactaceous plants were described in Brazil. Cactus virus X was reported from different cacti and is widely spread in the country (4). Three viruses infecting Opuntia tuna (Zygocactus virus X [ZyVX]), Hylocereus undatus (ZyVX and Schlumbergera virus X [SchVX]), and Schlumbergera truncata (Opuntia virus X [OpVX], ZyVX, and SchVX) were more recently identified based on partial nucleotide sequences of the RdRp gene in Sao Paulo State (5). We identified a new SchVX isolate from prickly pear (SchVX-Palma-PE) in Brazil. Plants collected in Pernambuco State were propagated and cultivated in the greenhouse. Total RNA from symptomless plants was extracted with an RNeasy Plant minikit and used for whole-transcriptome shotgun sequencing, using an Illumina HiSeq 2000 platform at Fasteris (Switzerland), aiming to study water-stress-related genes. *De novo* assembly was performed with the VELVET program (6) and in an initial BLAST search, several contigs were found to be potexvirus-derived. To further characterize these virus sequences, we used Geneious software to perform BLASTn and BLASTx searches against a viral reference sequence database. Comparisons of the nucleotide (nt) and the predicted amino acids (aa) sequences with other potexviruses were made with the SDT (7) and MUSCLE (8) programs. A contig of 6,615 nucleotides was identified as Schlumbergera virus X with an identity of 94% for the entire genome sequence with SchVX-K11 (GenBank accession no. AY366207). SchVX-K11 was first described infecting Schlumbergera bridgesii from a botanical garden in Croatia (former Yugoslavia) during the 1960s (9), but only in 2004 was it classified as a distinct *Potexvirus* member based on its nucleotide sequence (10).

The annotation of this contig rendered five complete open reading frames (ORFs). ORF1 (4,638 nt) encodes the RNA-polymerase (RdRp) with 1,545 amino acids. The triple gene block (TGB) is formed by ORF2 (690 nt) with 229 amino acids, ORF3 (333 nt) encoding a 110-aa protein, and ORF4 (183 nt) with 60 amino acids. The TGB proteins are involved in cell-to-cell movement of viral RNA. ORF5 (678 nt) encodes the coat protein (CP) with 225 amino acids. The 5′ untranslated region (UTR) is 83 nt long, and the 3′ UTR has 102 nt, excluding the poly(A) tail.

A phylogenetic analysis using the six complete potexvirus genomes from cactaceous hosts available in GenBank confirmed that the potexvirus isolated from prickly pear is an SchVX isolate. The comparison of the partial sequence of the RdRp gene with the sequences of SchVX previously identified in Brazil (5) revealed an identity of 94% with the isolate from H. undatus and 86% with the isolate from *S. truncate*.

### Nucleotide sequence accession number.

The nucleotide sequence of SchVX-Palma-PE has been deposited in GenBank under the accession number KP090203.
